# Prevalence of *Chlamydia trachomatis* and *Neisseria gonorrhoeae* infections and associated risk factors among pregnant women and key populations in Kenya: A multi-centre cross-sectional study

**DOI:** 10.1371/journal.pgph.0005479

**Published:** 2026-02-24

**Authors:** Catherine Ngugi, Borna A. Nyaoke, Leonard Kingwara, Pacific Akinyi, Mildred Mmbone, Thaddaeus W. Egondi, George M. Nyangweso, Helen Broadhurst, Gabrielle Kornmann, Rashmi Mathur, Esther Bettiol

**Affiliations:** 1 Ministry of Health of Kenya, Nairobi, Kenya; 2 Drugs for Neglected Diseases initiative (DNDi), Nairobi, Kenya; 3 Kenya National Public Health Institute, Ministry of Health of Kenya, Nairobi, Kenya; 4 Plus Project, Knutsford, United Kingdom; 5 Global Antibiotic Research & Development Partnership (GARDP), Geneva, Switzerland; University of Alabama at Birmingham, UNITED STATES OF AMERICA

## Abstract

Sexually-transmitted pathogens *Chlamydia trachomatis* (CT) and *Neisseria gonorrhoeae* (NG) cause curable but often asymptomatic bacterial infections. Missed diagnoses and treatment, leading to chronic infections, can cause clinical complications and increase transmission. Accurate prevalence estimates are essential for the effective public health control of these sexually transmitted infections, especially in Africa, where data are scarce. This cross-sectional study was undertaken across five sites in Nairobi, Mombasa, and Homabay in Kenya. Between February and July 2022, vaginal, urethral, or rectal swab samples were collected in pregnant women and populations at higher risk of sexually transmitted infections (key populations) aged ≥15 years and analysed using an NG/CT nucleic acid amplification test. The primary objective was to determine the prevalence of NG and CT in pregnant women and key populations in Kenya to provide input in developing national prevalence estimates. Multivariate regression analysis examined infection status based on participants characteristics. NG prevalence was significantly lower among pregnant women [1.0% (95%CI: 0.5-1.9)] compared to key populations [9.4% (95%CI: 6.9-12.5)], while CT prevalence was similar between the two populations: 9.6% (95%CI: 7.8-11.7) in pregnant women and 11.2% (95%CI: 8.4-14.5) in key populations. NG and CT prevalence were highest among younger individuals, reaching 5.7% and 15.1% in pregnant women <20 years and 25.0% and 50.0% in key populations <20 years, respectively. Prevalence of both pathogens decreased with increasing age. These findings support the development of national prevalence estimates, which will support better management of NG and CT infections in Kenya by directing preventive and control measures.

## Introduction

Sexually transmitted infections (STIs) have a large impact on global health [1]. The World Health Organization (WHO) estimates that, in 2020, there were 374 million new infections with one of four curable STIs (chlamydia, gonorrhoea, syphilis, and trichomoniasis) [2]. This is despite many prevention and control efforts [3]. Bacterial infections caused by *Chlamydia trachomatis* (CT) and *Neisseria gonorrhoeae* (NG) are both curable with appropriate antibiotics. While CT and NG STIs can cause urethritis in men and symptomatic cervicitis in women, the frequent absence of symptoms can lead to sustained infections and further transmission to partners [4,5]. Therefore, timely screening and treatment are important. Moreover, serious complications such as pelvic inflammatory disease can result, if untreated, in tubal scarring and infertility, life-threatening ectopic pregnancy, and chronic pelvic pain [4]. Disseminated gonorrhoea can even cause infectious arthritis and other serious complications [5]. In addition, infection with NG may enhance transmission of HIV by 2- to 5-fold [6]. Untreated individuals can also transmit the infection to others, including to newborns during delivery, which increases the risk of developing conjunctivitis or serious, life-threatening infections [7,8].

Accordingly, the WHO developed a Global health sector strategy on STIs [9]. Priority actions for countries include the estimation of the prevalence and incidence of STIs at the national level, to enable advocacy, planning, and impact monitoring of national STI public health response. The WHO estimated that, in 2020, there were 82 million people aged 15–49 who were newly infected with NG and 129 million people newly infected with CT [2]. Evidence suggests that NG may be decreasing in prevalence over time [10]. For CT, the situation is not improving; the age-standardized incidence rates from the 2019 Global Burden of Disease study show a yearly 0.29% increase worldwide [11].

However, the global burden of NG and CT infections, as estimated by their prevalence and the monitoring of their prevalence trends over time, are difficult to determine as screening (either in routine or for point prevalence) is limited by its cost and availability, especially in low-resource settings like Africa. The most recent worldwide data were collected in 2020 [2]. In addition, multi-drug and extensive drug resistance is becoming a concern for the treatment of gonococcal infections, including in Kenya [12,13]. Tackling this problem requires knowledge of the prevalence of NG and CT in both the general population and among populations at higher risk of STIs (key populations [14]), such as sex workers, and men who have sex with men (MSM). Even so, current monitoring of NG and CT is suboptimal worldwide [15,16]. Methods such as the statistical trend-fitting Spectrum-STI model have helped estimate prevalence in certain countries [17]. Accordingly, the WHO provided a standard protocol to assess the prevalence of gonorrhoea and chlamydia among pregnant women in antenatal care (ANC) clinics as a method of providing proxy estimates of the prevalence in the general population [18].

Africa generally reports high rates of NG and CT infections [10,15,16]. The worldwide prevalence of gonorrhoea in pregnant women was estimated at 1.85%, with the highest rate in the African region (3.53%) [10]. Similarly, the global prevalence of CT among females worldwide was 3.1% compared to 3.8% for females in Africa [16]. In addition, adolescents are also disproportionately affected by STIs [1]. This highlights the urgent need for more accurate national prevalence estimates globally and in Africa [10,15]. In Kenya, most pregnant women use ANC clinic services at least once during their pregnancies. Key populations use dedicated clinics such as the Drop-in Centre (Dice) clinics to minimize the stigma and discrimination encountered when seeking health care. Therefore, the aim of this cross-sectional study was to determine the prevalence of NG and CT in pregnant women, as proxy of the general population, and in key populations to estimate national NG and CT prevalence in Kenya.

## Methods

### Ethics statement

The study received National Ethics approval from the Kenyatta National Hospital-University of Nairobi (KNH-UoN) Ethics Review Committee (REF: P667/08/2021) and a National Research License (REF: 328194) from The National Commission for Science, Technology, and Innovation (NACOSTI) for Mombasa, Nairobi and Homabay counties. Mombasa County received additional approval from the Coast General Teaching and Referral Hospital Ethics Review Committee (REF: ERC-CGH/MSc/VOL.I). Nairobi County received additional approval from the Nairobi Metropolitan Services- Health Directorate’s Research Technical Working Group (REF: EOP/NMS/HS/096). Homabay County relied on the Ministry of Health Letter of Support and National ERC and NACOSTI approvals. There were no deviations from the study protocol after approval was obtained. Formal consent was obtained from all participants as detailed in the Procedures section below.

### Study design

This was a cross-sectional study carried out in five sites in three different counties in Kenya between 9^th^ February 2022 and 22^nd^ July 2022. The three antenatal care (ANC) centres (with participants recruitment period at each site) were Mbagathi Health Centre in Nairobi (1^st^ March -12^th^ July 2022), Coast Provincial General Hospital in Mombasa (8^th^ April – 14^th^ July 2022) and Homa-Bay County Referral Hospital in Homabay (9^th^ February – 14^th^ July 2022). The two Drop-in Centre (Dice) clinics were Special Treatment Centre Casino Health Centre in Nairobi (1^st^ March – 22^nd^ July 2022) and International Centre for Reproductive Health in Mombasa (11^th^ April – 13^th^ July 2022). The study was conducted by the Ministry of Health (MOH) in Kenya, in collaboration with GARDP and Drugs for Neglected Diseases initiative (DNDi).

### Participants

There were two distinct populations included in the study: pregnant women who attended the ANC centres and populations at higher risk of STIs (key populations), which included female sex workers (FSW), MSM, and MSM sex workers (MSM-SW) who attended Dice clinics. All consecutive participants who fulfilled the eligibility criteria and provided written consent were included (Table 1).

**Table 1:** Inclusion and exclusion criteria for pregnant women and key populations.

Criteria	Pregnant women	Key populations
**Inclusion criteria**	Pregnant women attending the ANC centre	Key populations participants attending the Dice clinic
	Aged ≥15 years	
	Willingness and ability to provide a laboratory specimen	
	Willingness and ability to be contacted by study staff and to return to the clinic for treatment if required	
	Willingness and ability to provide informed consent/assent	
**Exclusion criteria**	Previously enrolled in the study	
	Pregnant women with a high risk of abortion, i.e., recurrent pregnancy loss, threatened abortion, history of cervical conisation	
	For females: Use of any systemic or intravaginal antibiotics with activity against NG and CT within 30 days prior to screening	

ANC = Antenatal care; CT = *Chlamydia trachomatis*; NG = *Neisseria gonorrhoeae*.

### Procedures

On the day of the first visit, after adequate presentation of aims, methods, anticipated benefits, and potential hazards of the study the Investigator or designee had the responsibility to obtain written consent from each participant before any study-related procedures taking place. If the participant was unable to read or write, a literate witness (who was to have no connection to the research team or the Sponsor, and, if possible, was to be selected by the participant) was required to sign the form. The Investigator was also required to obtain the assent of children (< 18 years), but their assent had to be completed by the consent of a parent or guardian. Minors < 18 years who were considered emancipated minors (e.g., < 18 years and married) were able to give and sign informed consent form (ICF) to participate in the study. A new ICF was obtained in participants who were recruited as minors and that reached their 18th birthday while still in the study. The written informed consent and assent documents were translated into the local language (Swahili). After informed consent/assent was obtained, the site staff collected socio-demographic, pregnancy, sexual, and STI history data through participant interviews. Afterwards, either a vaginal, urethral, or rectal swab sample was collected for each participant in the pelvic examination room according to local specimen collection procedures by a designated healthcare practitioner and based on sexual behaviour history. The specimen was used for the CT/NG nucleic acid amplification test (NAAT) diagnostic test (Cepheid GeneXpert CT/NG Assay kit) at the microbiology laboratory of the selected sites.

Participants testing positive for CT or NG were confidentially notified of their results by telephone by the investigator or nursing staff. The participants who tested positive for NG and/or CT and their partners were offered treatment as per the Kenyan national standard of care for STI treatment based on WHO 2021 guidelines [19]. Adverse events related to study participation (in particular to sample collection) were collected up to 30 days after the initial visit.

### Study objectives

The primary objective was to determine the prevalence of NG and CT in pregnant women and key populations in Kenya to provide input in developing national prevalence estimates.

As a secondary objective, a sub-analysis of the NG and CT infection status according to different socio-demographic, pregnancy-related, sexual, and STI history-related data was performed to determine if there is heterogeneous prevalence across different groups in Kenya.

### Sample size

The sample sizes were determined using unpublished estimates of gonorrhoea prevalence in Kenya calculated with the Spectrum-STI tool [17], using previously published data: For pregnant women, an expected prevalence of 1.04% was used to determine sample size, while for key populations, an expected prevalence of 2.08% among FSW as proxy for key populations was used. Other parameters used included a 5% significance level and a degree of precision for the prevalence estimate set at 67% of the prevalence estimate (0.7% margin of error). The sample size was adjusted to account for withdrawal or missing test results, assumed to be approximately 10%. The study target sample size was, therefore, 904 for pregnant women and 448 for the key populations.

### Statistical methods

Descriptive summary statistics are provided using mean and standard deviation (SD) or median, and range for continuous variables and numbers with relative proportions by category. The prevalence of NG and CT with a 95% exact confidence interval (95%CI) was calculated and reported using the Clopper-Pearson method. Unadjusted prevalence and prevalence ratio (PR) of NG or CT and corresponding 95% CI baseline characteristics were calculated for the following variables: for pregnant women: residence, region, age, trimester of pregnancy and STI history) and for the key populations: residence, region, age, type of key populations and STI history. The adjusted prevalence and PR of NG or CT infection and corresponding 95%CI were also computed using a standard multivariate logistic regression analysis using the same variables. All data summarisation and analyses were performed using SAS version 9.4.

### Inclusivity in global research

Additional information regarding the ethical, cultural, and scientific considerations specific to inclusivity in global research is included in the Supporting Information (S1 Checklist).

## Results

**Fig 1:**
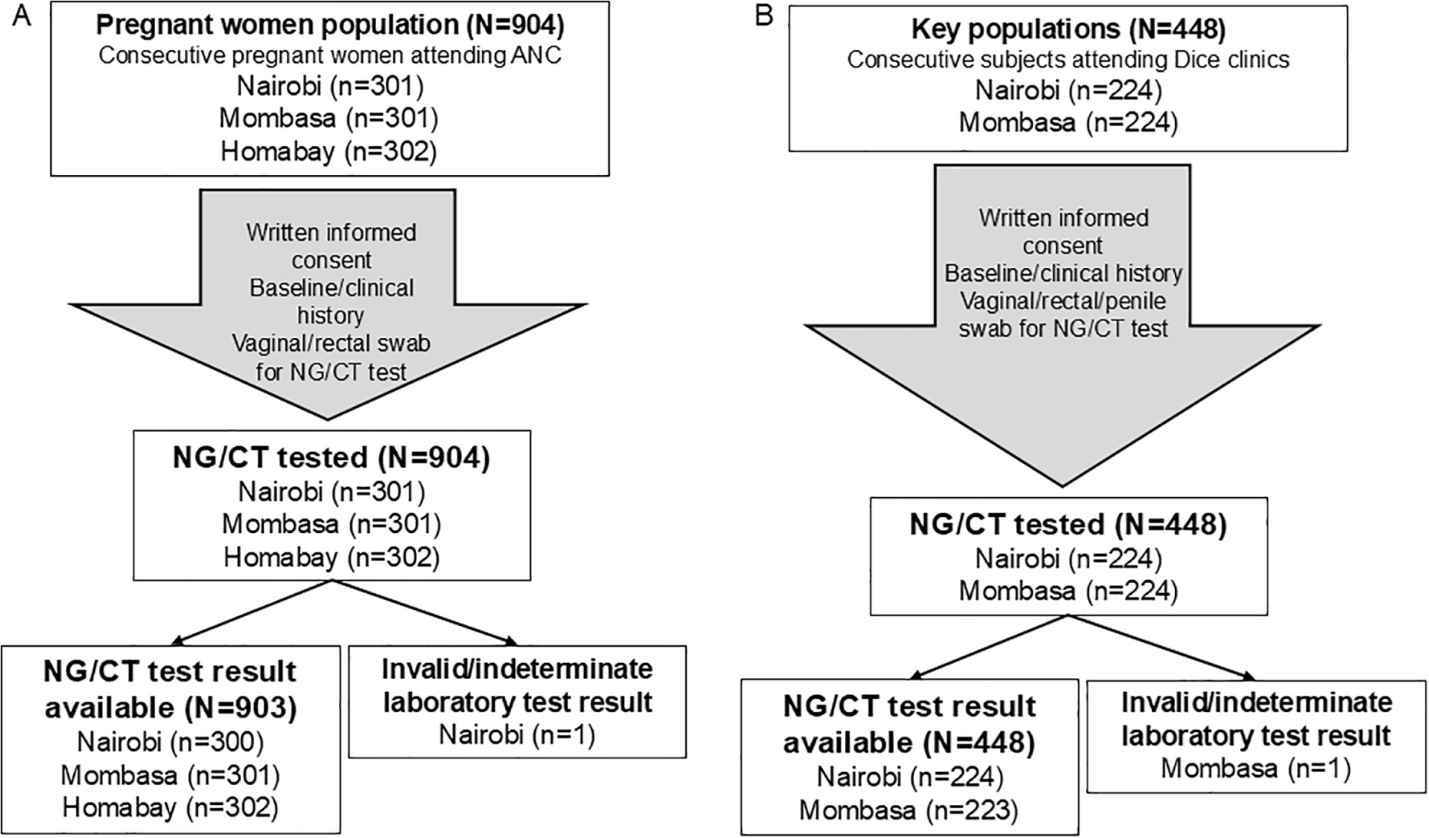
Participants flow diagram. A. Pregnant women were included from ANC clinics in three locations. B. Key populations were included from Dice clinics. ANC: antenatal care; CT: *Chlamydia trachomatis*; Dice: Drop-in Centre; NG: *Neisseria gonorrhoeae*.

Of the 1,352 individuals enrolled (904 pregnant women and 448 key populations) over a 5 months period, 1'352 were tested for CT and NG and 1.350 contributed to the prevalence estimates. Details of the enrolment and testing of the study populations are shown in the flow diagram in Fig 1. In terms of specimen collected, 1131/1352 females had a vaginal swab and 1/1132 female had a rectal swab. For males, 181/ 215 males had a urethral swab and 34/215 males had a rectal swab, while 2/5 transgender women had a urethral swab and 3/5 had a rectal swab.

The baseline characteristics of the study populations are shown in Table 2 and by study site in S1 Table. The age of the pregnant women ranged between 16–47 years. In the key populations, the age ranged between 17–62 years. A large majority of participants were of Kenyan nationality (>95%), with more residents in urban versus rural areas. More pregnant women had completed secondary or higher education than the key populations (80% versus 47%). Around 39% of the pregnant women were unemployed. In the key populations, only 2.2% were unemployed.

**Table 2:** Demographic characteristics of pregnant women and key populations in Kenya, February-July 2022.

Characteristic	Pregnant women (N = 904)	Key populations (N = 448)
**Age (years)**		
Mean ±SD	26.9 ± 5.6	31.0 ± 7.5
Min – Max	16–47	17–62
**Nationality [n (%)]**		
Kenyan	896 (99.1)	438 (97.8)
Non-Kenyan	8 (0.9)	10 (2.2)
**Residence [n (%)]**		
Urban	628 (69.5)	358 (79.9)
Rural	276 (30.5)	90 (20.1)
**Education level [n (%)]**		
No education	3 (0.3)	2 (0.4)
Some primary education	49 (5.4)	92 (20.5)
Completed primary education	123 (13.6)	63 (14.1)
Some secondary education	91 (10.1)	82 (18.3)
Completed secondary education	308 (34.1)	107 (23.9)
Some tertiary education	73 (8.1)	50 (11.2)
Completed tertiary education	257 (28.4)	52 (11.6)
**Occupation [n (%)]**		
Salaried	154 (17.0)	45 (10.0)
Wage-earner	52 (5.8)	213 (47.5)
Self-employed	263 (29.1)	162 (36.2)
Unemployed	350 (38.7)	10 (2.2)
Student	85 (9.4)	18 (4.0)

SD = Standard deviation.

The key populations’ specific characteristics are shown in Table 3, with site-specific data in S2 Table. About half of the key populations (50.9%) were FSW, while 43.5% were MSM and 5.6% MSM-SW. The median (min-max) number of reported sexual partners in the previous month was 2.0 (0-60).

**Table 3:** Baseline characteristics of key populations in Kenya, February-July 2022.

Characteristic	Key populations (N = 448)
**Sex [n (%)]**	
Male	215 (48.0)
Female	228 (50.9)
Transgender women	5 (1.1)
**Type of key populations [n (%)]**	
FSW	228 (50.9)
MSM	195 (43.5)
MSM-SW	25 (5.6)
**Sexual behaviour in the previous month [n (%)]**	
Participants who reported their sexual behaviour	332 (74.1)
**Number of sexual partners**	
Median (Min – Max)	2.0 (0–60)

SD = standard deviation; FSW = female sex worker; MSM = men who have sex with men; MSM-SW = men who have sex with men who sell sex.

Obstetric history is shown in Table 4, with site data in S3 Table. A large majority of the pregnant women were in the second and third trimesters of pregnancy. About a third were in their first pregnancy.

**Table 4:** Obstetrical history of pregnant women in Kenya, February-July 2022.

Characteristic	Pregnant women (N = 904)
**Gestational age (weeks)**	
Mean ±SD	26.7 ± 8.3
Min – Max	4–42
1st trimester [n (%)]	84 (9.3)
2nd trimester [n (%)]	401 (44.4)
3rd trimester [n (%)]	419 (46.3)
**Number of pregnancies including the current one (mean ±SD)**	2.3 ± 1.4
**Number of women with a previous live birth [n (%)]**	526 (58.2)
**Number of women in their first pregnancy** **(including current one) [n (%)]**	326 (36.1)
**Number of live births (mean ±SD)**	1.1 ± 1.2
**Number of women with pregnancy loss [n (%)]**	146 (16.2)
**Number of pregnancy loss (mean ±SD)**	0.2 ± 0.5

SD = Standard deviation.

The history of previous STIs in the past 12 months of the two populations is shown in Table 5, with site-specific information in S4 Table. In pregnant women, 35/869 (3.9%) participants reported a history of previous STI, the most frequent being HIV or acquired immunodeficiency syndrome (AIDS) (26/35, 74.3%). In the key populations, 89/359 (19.9%) participants reported having had a history of STI, most frequently HIV/AIDS (59/89, 65.2%).

**Table 5:** Rate and type of reported sexually transmitted infections (STIs) in the past 12 months in pregnant women and key populations in Kenya, February-July 2022.

	Pregnant women	Key populations
**Participants reporting a history of STI in the past 12 months [n/N (%)]**		
**Yes**	35/904 (3.9)	89/447 (19.9)*
**No**	869/904 (96.1)	358/447 (79.9)
**Type of reported previous STI [n/N (%)]**		
HIV/AIDS	26/35 (74.3)	59/89 (65.2)
Syphilis	2/35 (5.7)	2/89 (2.2)
Gonorrhoea	--	9/89 (10.1)
Genital herpes	--	1/89 (1.1)
STI (not specified)	7/35 (20.0)	19/89 (21.4)

AIDS = acquired immunodeficiency syndrome; HIV = human immunodeficiency virus; STI = sexually transmitted infection.

* 1/448 participant STI history status in the key populations was unknown.

Note: ‘STI (not specified)’ indicates participants who reported having had an STI in the past 12 months but did not specify or recall the particular type.

The overall unadjusted prevalence of NG and CT is shown in Table 6. Nine of 903 pregnant women tested positive for NG (prevalence: 1% [95%CI: 0.5-1.9]) and 87 tested positive for CT (prevalence: 9.6% [95%CI: 7.8-11.7]). Furthermore, 10.3% (95%CI: 8.4-12.5) of the pregnant women had an infection with NG and/or CT, and 0.3% (95%CI: 0.1-1.0) had a CT/NG coinfection. In the key populations (n = 447), 42 participants tested positive for NG (prevalence: 9.4% [95%CI: 6.9-12.5]), while 50 tested positive for CT (prevalence of 11.2% (95%CI: 8.4-14.5). Of these, 18.6% (95%CI: 15.1-22.5) tested positive for NG and/or CT, while 2.0% (95%CI: 0.9; 3.8) had a CT/NG coinfection (Table 6).

**Table 6:** Overall unadjusted prevalence of NG and CT in pregnant women and key populations in Kenya, February-July 2022.

STI	Pregnant women (N = 903)		Key populations (N = 447)	
	n (%)	95%CI	n (%)	95%CI
**NG**	9 (1.0)	0.5–1.9	42 (9.4)	6.9–12.5
**CT**	87 (9.6)	7.8–11.7	50 (11.2)	8.4–14.5
**NG and/or CT**	93 (10.3)	8.4–12.5	83 (18.6)	15.1–22.5
**NG and CT coinfection**	3 (0.3)	0.1–1.0	9 (2.0)	0.9–3.8

CT = *Chlamydia trachomatis*; CI = confidence interval; NG = *Neisseria gonorrhoeae*; STI = sexually transmitted infection.

When looking at the prevalence of NG in pregnant women based on different demographic characteristics, the prevalence of NG was higher in younger pregnant women, with 5.7% in those <20 years versus 1% in those aged 20–29 years versus none in older women (Table 7). Due to the low number of pregnant women who tested positive for NG in the age subgroups, it was not possible to assess the PR or do multivariate logistic regression analysis for NG. For CT, multivariate logistic regression analysis showed that the PR for CT (both unadjusted and adjusted) show a trend indicating that lower age is associated with a higher risk of CT infections. For women aged 30–39, the 95%CI of unadjusted and adjusted PR are below 1, showing that this subgroup is less likely to be infected with CT than those in the 20–29 age group (e.g., adjusted PR = 0.42, 95%CI: 0.23-0.78). The other demographics factors did not influence the prevalence of NG or CT infection. In the unadjusted PR analysis, living in a rural area was associated with a lower prevalence of CT infection than living in urban areas (PR = 0.59, 95%CI: 0.36-0.98); however, the adjusted analysis did not confirm this trend.

**Table 7:** Prevalence and prevalence ratios for NG and CT according to demographics in pregnant women in Kenya, February-July 2022.

Variable	NG				CT			
	Unadjusted prevalence (95%CI)	Unadjusted PR (95%CI)	Adjusted prevalence (95%CI)	Adjusted PR (95%CI)	Unadjusted prevalence (95%CI)	Unadjusted PR (95%CI)	Adjusted prevalence % (95%CI)	Adjusted PR (95%CI)
**Residence**								
Rural (n = 628)	1.1 (0.2–3.1)	1.14 (0.29–4.51)	*	*	6.5 (3.9–10.1)	0.59 (0.36–0.98)	7.2 (4.3–12.3)	0.79 (0.41–1.51)
Urban (n = 276)	1.0 (0.4–2.1)	Ref	*	*	11.0 (8.7–13.7)	Ref	9.2 (6.9–12.3)	Ref
**Region**								
Homabay (n = 302)	1.7 (0.5–3.8)	Ref	*	*	11.3 (7.9–15.4)	Ref	9.5 (6.6–13.7)	Ref
Mombasa (n = 301)	0.3 (0.0–1.8)	0.20 (0.02–1.71)	*	*	6.3 (3.8–9.7)	0.56 (0.33–0.96)	6.8 (4.1–11.2)	0.71 (0.37–1.37)
Nairobi (n = 301)	1.0 (0.2–2.9)	0.60 (0.15–2.50)	*	*	11.3 (8.0–15.5)	1.01 (0.64–1.58)	9.8 (6.7–14.4)	1.03 (0.65–1.63)
**Age**								
> 20 (n = 53)	5.7 (1.2–15.7)	*	*	*	15.1 (6.7–27.6)	1.33 (0.68–2.62)	13.0 (6.7–25.1)	1.20 (0.60–2.37)
20–29 (n = 591)	1.0 (0.4–2.2)	*	*	*	11.4 (8.9–14.2)	Ref	10.9 (8.6–13.8)	Ref
30–39 (n = 240)	0	*	*	*	4.6 (2.3–8.1)	0.40 (0.22–0.75)	4.6 (2.6–8.2)	0.42 (0.23–0.78)
40–49 (n = 20)	0	*	*	*	5.0 (0.1–24.9)	0.44 (0.06–3.01)	4.4 (0.6–29.9)	0.41 (0.06–2.78)
≥ 50 (n = 0)	–	*	*	*	–	–	–	–
**Trimester**								
1 (n = 84)	1.2 (0.0–6.5)	1.66 (0.17–15.75)	*	*	7.1 (2.7–14.9)	0.77 (0.33–1.75)	6.3 (2.9–13.7)	0.73 (0.32–1.67)
2 (n = 401)	1.2 (0.4–2.9)	1.74 (0.42–7.22)	*	*	10.5 (7.7–13.9)	1.12 (0.74–1.70)	9.1 (6.7–12.4)	1.06 (0.70–1.60)
3 (n = 419)	0.7 (0.1–2.1)	Ref	*	*	9.3 (6.7–12.5)	Ref	8.6 (6.3–11.8)	Ref
**History of STI in the past 12 months**								
No (n = 868)	0.9 (0.4–1.8)	Ref	*	*	9.8 (7.9–12.0)	Ref	8.8 (7.0–11.0)	Ref
Yes (n = 35)	2.9 (0.1–14.9)	3.10 (0.40–24.11)	*	*	5.7 (0.7–19.2)	0.58 (0.15–2.28)	5.0 (1.3–19.2)	0.57 (0.15–2.22)

Adjusted PR from logistic regression. Adjusted PR was only computed for participants with NG or CT, where the number of cases was sufficient for model convergence.

* Indicates where the model did not converge due to the sparse number of cases in some subgroups.

CT = *Chlamydia trachomatis*; CI = confidence interval; NG = *Neisseria gonorrhoeae*; PR = prevalence ratio; Ref = reference prevalence.

In the key populations, the prevalence of both pathogens was highest in the < 20 years age group (Table 8). The PR for NG (both unadjusted and adjusted) show a trend indicating that a lower age was associated with a higher risk of NG infection. For CT, both the unadjusted and adjusted PR show that age < 20 years was strongly associated with CT infection compared to the 20–29 age group (unadjusted PR = 4.12, 95%CI: 2.10-8.09; adjusted PR = 3.61, 95%CI: 1.84-7.06). However, the < 20 years age group was small (n = 12), and the estimate would be highly impacted by a small difference in group size. Regarding the impact of STI history, an opposite effect was observed: having a history of STI in the previous 12 months was strongly associated with a higher prevalence of NG (Unadjusted PR = 2.23, 95%CI: 1.24-4.02), but associated with a lower prevalence of CT (Unadjusted PR = 0.45, 95%CI: 0.18-10.9). Regarding other influencing factors, there were some differences in prevalence by residence and by type of key populations. However, the CI overlapping with 1 prevented to conclude that these factors had a strong association with either NG or CT prevalence.

**Table 8:** Prevalence and prevalence ratios for NG and CT according to demographics in the key populations in Kenya, February-July 2022.

Variable	NG				CT			
	Unadjusted prevalence (95%CI)	Unadjusted PR (95%CI)	Adjusted prevalence (95%CI)	Adjusted PR (95%CI)	Unadjusted prevalence (95%CI)	Unadjusted PR (95%CI)	Adjusted prevalence (95%CI)	Adjusted PR (95%CI)
**Residence**								
Rural (n = 358)	6.7 (2.5–13.9)	0.66 (0.29–1.52)	*	0.90 (0.34–2.35)	7.8 (3.2–15.4)	0.65 (0.30–1.39)	*	0.54 (0.24–1.22)
Urban (n = 90)	10.1 (7.2–13.7)	Ref	*	Ref	12.0 (8.9–15.9)	Ref	*	Ref
**Region**								
Mombasa (n = 224)	7.2 (4.2–11.4)	0.62 (0.34–1.12)	*	0.69 (0.35–1.39)	11.7 (7.8–16.6)	1.09 (0.65–1.84)	*	1.21 (0.71–2.07)
Nairobi (n = 224)	11.6 (7.7–16.5)	Ref	*	Ref	10.7 (7.0–15.5)	Ref	*	Ref
**Age**								
< 20 (n = 12)	25.0 (5.5–57.2)	2.15 (0.75–6.13)	*	2.52 (0.87–7.34)	50.0 (21.1–78.9)	4.12 (2.10–8.09)	*	3.29 (1.68–6.47)
20–29 (n = 207)	11.7 (7.6–16.8)	Ref	*	Ref	12.1 (8.0–17.4)	Ref	*	Ref
30–39 (n = 169)	7.7 (4.2–12.8)	0.66 (0.35–1.26)	*	0.68 (0.35–1.30)	8.9 (5.1–14.2)	0.73 (0.40–1.34)	*	0.68 (0.37–1.24)
40–49 (n = 52)	3.8 (0.5–13.2)	0.33 (0.08–1.35)	*	0.37 (0.09–1.54)	7.7 (2.1–18.5)	0.63 (0.23–1.74)	*	0.57 (0.21–1.58)
≥ 50 (n = 8)	0	0	*	0	0	0	*	0
**Type of key populations**								
FSW (n = 228)	8.8 (5.5–13.3)	Ref	*	Ref	12.3 (8.4–17.3)	Ref	*	Ref
MSM (n = 195)	9.2 (5.6–14.2)	1.05 (0.57–1.92)	*	0.95 (0.52–1.76)	10.8 (6.8–16.0)	0.87 (0.51–1.49)	*	0.87 (0.52–1.46)
MSM-SW (n = 25)	16.0 (4.5–36.1)	1.82 (0.67–4.89)	*	1.53 (0.56–4.22)	4.0 (0.1–20.4)	0.32 (0.05–2.28)	*	0.39 (0.05–2.79)
**History of STI in the past 12 months**								
No^§^ (n = 359)	7.5 (5.0–10.8)	Ref	*	*	12.6 (9.3–16.5)	Ref	*	Ref
Yes (n = 89)	16.9 (9.8–26.3)	2.23 (1.24–4.02)	*	*	5.6 (1.8–12.6)	0.45 (0.18–1.09)	*	0.50 (0.20–1.27)

Adjusted PR from logistic regression. Adjusted PR was only computed for participants with NG or CT, where the number of cases was sufficient for model convergence.

* Indicates where the model did not converge due to the sparse number of cases in some subgroups.

CT = *Chlamydia trachomatis*; CI = confidence interval; FSW = female sex worker; MSM = men who have sex with men; MSM-SW = men who have sex with men who sell sex; NG = *Neisseria gonorrhoeae*; PR = prevalence ratio; Ref = reference prevalence.

§ One participant’s STI history was unknown and was included in the No category.

Due to the small subgroup size and/or the low number of infections in total or per subgroup, it was not possible to obtain logistic regression model convergence or performance for all variables, as noted in Table 7 and Table 8.

The treatments received by the participants who tested positive for NG and/or CT during the study are summarised in S5 Table for pregnant women and S6 Table for key populations. Overall, 94.6% (88/93) of pregnant women who had tested positive for NG and/or CT received treatment. Five out of 93 were lost to follow up and did not receive treatment as part of this study despite numerous attempts to contact them (2 had tested positive for NG and 3 for CT). In the key populations, 89.2% (74/83) of participants received treatment. Nine were lost to follow up (3 had tested positive for NG, 4 for CT and 2 had a co-infection). There were no adverse events related to study participation reported during the trial study period.

The analysis of the prevalence and PR for combined STIs (NG and/or CT) infection according to demographics is provided in S7 Table for pregnant women and S8 Table for key populations. These results also suggest that age is the only factor with a relationship to the STI rate in this study.

## Discussion

The aim of this study was to determine the prevalence of NG and CT in pregnant women and key populations in Kenya to provide input in developing national prevalence estimates. Kenya is a large country with a population of around 53.7 million living in 47 counties, with major differences in population densities. Homa Bay County is a rural neighbourhood in Western Kenya on the shores of Lake Victoria that is largely inhabited by agricultural and fishing communities, but overall the Western area has the highest HIV prevalence in Kenya [20] with a significant MSM population due to the urban environment and transportation hubs [21]. Nairobi, the capital city of Kenya, constitutes its own county within Kenya and has a mid-level STI prevalence, with large FSW and MSM populations [22]. Meanwhile, the Eastern coastal region of Kenya has a large proportion of people who use drugs compared to the rest of the country, particularly in urban areas of coastal cities, like Malindi and Mombasa [22], and has a large population of MSM. We selected study sites in these three areas to cover different STI epidemiological zones and provide prevalence estimates that are as representative as possible.

Our study shows that the prevalence of NG was much lower in pregnant women as a proxy for the general population (1.0%, 95%CI: 0.5-1.9) than in key populations, who are at higher risk for contracting STIs (9.4%, 95%CI: 6.9-12.5). On the contrary, CT infection prevalence was almost as high in pregnant women (9.6%, 95%CI: 7.8-11.7) as in key populations (11.2%, 95%CI: 8.4-14.5). In addition, the multivariate regression analysis of NG and CT infections in each population was performed to identify risk factors for NG or CT infection. This analysis suggested that age might be a risk factor for both pregnant women and key populations, with younger participants in both groups having a higher prevalence of NG and CT infections.

The history of a previous STI was much higher in key populations (19.9%) than in pregnant women (3.9%), reflecting a higher exposure to sexually transmitted pathogens in higher-risk key populations, as expected. As pregnant women in Kenya are routinely screened for HIV and syphilis, these were the most common STIs reported. No pregnant woman reported a history of gonococcal, chlamydial, or herpes simplex infection in the past 12 months.

The comparison between the two populations in this study shows an important difference in NG prevalence, whereas the prevalence of CT remains relatively unchanged. In Kenya, STIs are managed syndromically, and routine screening for gonococcal and chlamydial infections is not performed, leading to under-detection. Moving from syndromic management to diagnostic-based clinical management, combined with routine screening programs – especially for key populations – would allow early detection of infections, limit complications, and promote antibiotic stewardship. The development of point-of-care tests that are easy, rapid, and affordable is a global public health priority [23] and a necessary step to enable routine screening in low- and middle-income countries in the future.

To assess prevalence trends over time, we compared our results with recently published meta-analyses and key studies published since 2010 in similar populations from Kenya. The global prevalence estimate for gonorrhoea was 0.9% in 2016 [24]. In 2021, a meta-analysis estimated the prevalence of NG was 3.3% in sub-Saharan Africa and 2.4% in Eastern Africa [25]. In Nyanza region of Kenya, the period prevalence of NG was 2% in 1221 HIV-negative women during pregnancy until 9 months postpartum in 2011–2013 [26]. In 2015, the NG prevalence was 1.0% (95%CI: 0.1-3.5) in 202 pregnant women attending ANC centres in Kilifi County Hospital [27]. Most recently, in 2014, 457 non-pregnant 18–34-year-old women from Kisumu who were screened for entry into a contraception study showed a 3.9% prevalence for NG [28]. Compared with the prevalence of NG reported in the present study (1.0%), this data suggests that the prevalence of NG in Kenyan pregnant women (as a proxy for the general population) has not increased.

The prevalence of CT among pregnant women reported in our study (9.6%) was higher than the 2016 global estimate of 3.8% [24] but similar to the 2022 prevalence derived from a meta-analysis, which was 10.8% for sub-Saharan Africa and 7.8% for Eastern Africa [25]. Moreover, between 2010 and 2021, data from seven single-centre/region studies that included between 200 and >1000 women (pregnant or non-pregnant) reported a prevalence for CT varying between 3.6% and 14.9% [26–32]. Overall, the prevalence of CT in the general Kenyan population appears to remain around 10%, which is higher than the 2016 global prevalence of 3.8%.

Prevalence trends for NG and CT in key populations are not readily available as no meta-analyses exist globally or in Africa. A systematic literature search by Whelan and colleagues concluded that there is much heterogeneity between studies, there is often overlap in the reporting of risk groups, and population-based prevalence estimates are very limited [15]. Previous prevalence estimates have been published in Kenyan FSW and MSM separately, most often in single-centre studies. For NG, between 2008 and 2022, data from four single-centre/region studies that included between 326 and 596 FSW were published [33–35], with NG prevalence varying between 1.1% and 6.9% vs 8.8% in the present study. For CT, in 2015, CT unadjusted prevalence was 2.8%, and the adjusted prevalence was 3.1% in 596 FSW from Nairobi [34], while in 2020–2021 the prevalence of CT was 14.1% in 449 young women on PEP, among which about a third were FSW [35] and in 2022, 6.3% of 379 FSW in Nairobi tested positive for NG [36].

Previously published studies on the prevalence of NG and CT infections in MSM in Kenya are scarce. This is, at least in part because same-sex relationships are illegal in Kenya. Some studies assessed STI prevalence in MSM using rectal specimen only: the prevalence in 81 MSM in coastal areas of Kenya in 2016–2017 was 13.5% for CT and 9.6% for NG [37], and the prevalence of combined NG and/or CT infection was 5.2% in 698 MSM in Kisumu in 2015–2016 [38]. More recently in 2021, an NG prevalence of 11.3% (95%CI: 6.1-16.5) was reported among 248 MSM in Nairobi [39], with a CT prevalence high at 51.0% (95%CI: 42.3-59.8) in the same study [39]. In our study where we collected in MSM specimens either from rectum or the urethra, we observed a relatively high prevalence in MSM of 9.2% for NG and 10.8% prevalence for CT. Nevertheless, there are large variations in prevalence between studies in key populations which suggest that more data is needed.

Looking at prevalence data from countries neighbouring Kenya published since 2018, there were no published studies from South Sudan or Somalia in either pregnant women or key populations. In Tanzania, a recent study in pregnant women attending ANC showed that 8.7% (95%CI: 6.2-12.2) tested positive for CT and 3.1% (95%CI: 1.7-5.5) for NG [40]. In Pemba Island, two studies in pregnant women were conducted and showed no NG infections [41,42], while for CT, in the first study, prevalence was 4.6% (95%CI: 2.8-6.9) [41] and in the other, at <20 weeks gestation, 9.1% (4/44) and at ≥20 weeks gestation, 6.1% (5/82) [42]. For Ethiopia, a systematic literature review and meta-analysis of prevalence of NG infections that included 12 studies including mostly participants with suspected STIs showed that the pooled estimate of NG prevalence was 20% (95%CI: 8–30) in that population [43]. For women, a prevalence of 7.6% (21/278) of NG infection was detected in women of childbearing potential attending a gynecology outpatient clinic in Northwestern Ethiopia [44]. Finally, a study in central Ethiopia where 580 pregnant women were tested for STIs showed 9.8% were positive for CT, and 4.3% for NG [45]. Published studies from Uganda were all conducted in high-risk or symptomatic populations. A cross-sectional study showed the prevalence of NG was 25% (20/79) and CT was 18% (14/79) in a mixed population of males and females attending outpatient STI clinics in Kampala [23]. STIPS was a large study which recruited 1825 participants in 2 cohorts (inland population and fishing population) in Southern Uganda [46]: in the inland populations, CT prevalence was 9.6% (95%CI: 7.9–11.7) and NG prevalence 5.0% (95%CI: 3.8–6.7), while in the fishing populations, CT prevalence was 9.9% (95%CI: 8.1– 12.0) and NG prevalence 8.4% (95%CI: 6.8–10.5). In men with urethral discharge recruited in Kampala, 66.8% (167/250) and 22.8% (57/250) tested positive for NG or CT, respectively [47], while a study in men living with HIV in rural southwestern Uganda showed 3/50 had CT (6%) and none had NG [48]. In women, among a cohort at risk for HIV who were planning a pregnancy and lived in rural southwestern Uganda, 13% had CT and 2% had NG infections [49] and among 220 FSW recruited in the Masaka region of Uganda, 1.3% were positiveNG and 2.6% for CT [50]. Overall, similarly to prevalence of CT and NG within Kenya over time, results from studies from neighbouring countries to Kenya show wide differences in prevalence of NG and CT infections depending on the population and the location.

Strengths of our study include that this is the first multi-centre study assessing NG and CT prevalence with a large sample size that included both pregnant women as proxy for the general population and key populations covering three different regions in Kenya. The recruitment duration (5 months) was within the recommended timeframe to evaluate point prevalence [18]. This should assist in guiding the development of national prevalence estimates for these two key STIs.

In terms of limitations, no adolescents below 16 years were included, and the sample sizes in the youngest and oldest age subgroups were smaller than the other subgroups. Due to low sample sizes, there was insufficient data in some of the categories for the multivariate regression analysis model to converge successfully, limiting data interpretation, and the regression analysis was not performed on all variables. Furthermore, even though the study was performed in 3 ANC centers and 2 Dice clinics, our prevalence data remain estimates for the entire Kenyan general population or key populations and it is challenging to know if the results are really generalisable. Using pregnant women as proxy for general population is recommended by WHO [18], but may not perfectly represent the general population. Moreover, pharyngeal prevalence of NG and CT was not assessed, which is a limitation, especially for NG, as it has been shown that pharyngeal infections plays an important role in NG transmission when left untreated [51] and the pharynx is a potential location for horizontal transmission of resistance genes in *Neisseria* spp. [52]. Finally, NG culture and antimicrobial susceptibility were not performed, restricting the evaluation of the prevalent strains in Kenya and their antimicrobial susceptibility profiles.

This study provides important data to estimate national NG and CT prevalence in the general and key populations in Kenya. Having national estimates will support better management of NG and CT infections in Kenya by directing preventive and control measures. Further studies are needed, particularly, in key populations and to evaluate the level of antimicrobial resistance as well in NG infection in Kenya.

## Supporting information

S1 Checklist Inclusivity in global research. (DOCX)

S1 Table Baseline characteristics of the study populations at each study location, February-July 2022. (DOCX)

S2 Table Key populations-specific baseline characteristics according to the Dice clinic location, February-July 2022. (DOCX)

S3 Table Obstetrical history of pregnant women according to the ANC clinic location, February-July 2022. (DOCX)

S4 Table History of STIs in the study populations at each study location, February-July 2022. (DOCX)

S5 Table Treatments given to pregnant women with positive NG and/or CT diagnostic tests at each ANC location, February-July 2022. (DOCX)

S6 Table Treatments given to key populations with positive NG and/or CT diagnostic tests at each Dice location, February-July 2022. (DOCX)

S7 Table Prevalence and prevalence ratios for NG and/or CT according to demographics in pregnant women in Kenya, February-July 2022. (DOCX)

S8 Table Prevalence and prevalence ratios for NG and/or CT according to demographics in the key populations in Kenya, February-July 2022. (DOCX)
