# Supplementary material for: Prevalence of Chlamydia trachomatis and Neisseria gonorrhoeae infections and associated risk factors among pregnant women and key populations in Kenya: A multi-centre cross-sectional study
Source: PLOS Glob Public Health. 2026 Feb 24;6(2):e0005479. doi: 10.1371/journal.pgph.0005479 (PMC12931752; doi:10.1371/journal.pgph.0005479)
Supplement: S1 Checklist — (DOCX) [file pgph.0005479.s001.docx]

Inclusivity in global research

PLOS’ policy on inclusivity in global research aims to improve transparency in the reporting of research performed outside of researchers’ own country or community and ensures that PLOS publications reporting global research adhere to high standards for research ethics and authorship. Authors of relevant research articles may be asked to complete the questionnaire below, which outlines ethical, cultural, and scientific considerations specific to inclusivity in global research. This questionnaire may be requested when researchers have travelled to a different country to conduct research, if research uses samples collected in another country, research with Indigenous populations or their lands, or if research is on cultural artefacts. Researchers travelling to another country solely to use laboratory equipment will not normally be required to complete the questionnaire. However, the questionnaire can be requested at the journal’s discretion for any submission – if you have been requested to complete this questionnaire by the PLOS journal you submitted to, please do so.

Please complete the questionnaire below and include this as a Supporting Information file with your manuscript. Note that if your paper is accepted for publication, this checklist will be published with your article in the supporting information files. Please ensure that you reference the checklist in the main body of your manuscript. We suggest adding a subsection ‘Inclusivity in global research’ to your Methods section and adding the following sentence: “Additional information regarding the ethical, cultural, and scientific considerations specific to inclusivity in global research is included in the Supporting Information (SX Checklist)”

The questions have been designed to be applicable to a wide range of study types, and there are subsections for both human subjects research and non-human subjects research. If any of the questions are not relevant to your research please mark them as “N/A” as appropriate.

**Ethical considerations, permits and authorship**

*This section is applicable to all research types.*

Provide details as to who granted permissions and/or consent for the study to take place in the Methods section of your manuscript. This should include the names of **all** ethics boards, governmental organizations, community leaders or other bodies that provided approval for the study. If individuals provided approval refer to these people by their role or title but do not list their name(s).

Reported in the methods section:

The study was conducted by the Ministry of Health (MOH) in Kenya, in collaboration The Global Antibiotic Research & Development Partnership (GARDP) and Drugs for Neglected Diseases initiative (DNDi). The study received National Ethics approval from the Kenyatta National Hospital-University of Nairobi (KNH-UoN) Ethics Review Committee (REF: P667/08/2021) and a National Research License (REF: 328194) from The National Commission for Science, Technology, and Innovation (NACOSTI) for Mombasa, Nairobi and Homabay counties. Mombasa County received additional approval from the Coast General Teaching and Referral Hospital Ethics Review Committee (REF: ERC-CGH/MSc/VOL.I). Nairobi County received additional approval from the Nairobi Metropolitan Services- Health Directorate’s Research Technical Working Group (REF: EOP/NMS/HS/096). Homabay County relied on the Ministry of Health Letter of Support and National ERC and NACOSTI approvals.

If there were any deviations from the study protocol after approval was obtained please provide details of these changes in the Methods section of your manuscript.

There were no deviations from the study protocol after approval was obtained and this is mentioned in the methods section.

Did this study involve local collaborators that are residents of the country where the research was conducted or members of the community studied? If you do not have any authors from said communities, please provide an explanation for this below.

Yes, the majority of the authors reside in Kenya where the research was conducted. The first author and principal investigator was Dr Catherine Ngugi from the Ministry of Health, Kenya. The county laboratories were coordinated by the Kenya National Public Health Institute, Kenya which is headed by Leonard Kingwara. Pacific Akinyi was the study coordinator in Kenya. Several collaborators from the DNDi Kenya office were also involved in the study (Borna A. Nyaoke, Mildred Mmbone, Thaddaeus W. Egondi, George M. Nyangweso).

Everyone listed as an author should meet PLOS’ criteria for authorship and all individuals who meet these criteria should be included in the author byline, rather than the acknowledgements. For further information please see the journal’s Authorship Policy.

**Human subjects research (e.g. health research, medical research, cross-cultural psychology)**

Did you obtain written informed consent from a representative of the local community or region before the research took place? How did you establish who speaks for the community? Details of written informed consent obtained from study participants should be reported separately in the Methods section of your manuscript.

The research took place in clinics (antenatal care and Dice clinics) and all participants signed informed consent forms as described in the manuscript Methods’ section. For ethics approval at the county’s hospital administration level, see first question of this checklist in Ethical considerations, permits and authorship Section.

How did members of the local community provide input on the aims of the research investigation, its methodology, and its anticipated outcome(s)?

This was done through continuous needs assessment surveys and interviews that the Ministry of Health, Kenya conducts in its ante-natal clinics and the Drop-in centres (Dice) which are community-based and specifically designed for key populations (like Adolescent Girls and Young Women and sex workers) to access confidential, non-discriminatory HIV prevention, treatment, and other integrated health services in a safe environment.

When engaging with the local community, how did you ensure that the informed consent documents and other materials could be understood by local stakeholders?

The informed consent documents were translated into Kiswahili ensuring patients and local stakeholders could engage adequately. Translated documents were back translated into English to verify that the original meaning was preserved and culturally appropriate.

Technical terms were replaced with simple, everyday language. Consent was treated as a conversation, not a formality, participants were encouraged to ask questions and express concerns.

Will the findings of the research be made available in an understandable format to stakeholders in the community where the study was conducted (e.g. via a presentation, summary report, copies of publications, etc.)? Please provide details of how this will be achieved.

Yes, the findings were made available to stakeholders in the community in formats that are clear, accessible, and culturally appropriate. The clinical study report was provided for review and finalization by the Ministry of Health Kenya and disseminated to Homabay, Nairobi and Mombasa County hospitals. Printed and digital briefs highlighting key statistics, implications, and recommended actions were also provided to the antenatal and Dice clinics.

Scientific publications will be made available online, with links shared via Ministry of Health platforms and partner NGOs.

On an individual participant level, laboratory results of the patients were provided as soon as received, management provided, and education provided on prevention of sexually transmitted infections.

**Non-human subjects research using specimens/ animals collected as part of the study, or those housed in archival collections. Examples include archaeology, paleontology, botany and zoology.**

Did the permission you obtained from a local authority to perform the study include an agreement on access to outputs and benefit sharing? This may include procedures to enable fair distribution of the benefits and resources arising from the research performed. Please include any details of Prior Informed Consent and Benefit Sharing Agreements obtained. These may be required by field-specific regulations, for example the Convention on Biological Diversity (CBD) and the associated Nagoya Protocol.

N/A

If the material used in your study was imported, please A) provide the year it was imported and B) indicate whether permits were obtained to import/export the materials used, C) provide details of any permits obtained. If this information is not available, please indicate this.

N/A

If you used archival specimens, please state how the material used in your study was acquired by the institute it is held in and provide details of any permits obtained for the original excavations/ sample collection. If this information is not available, please indicate this.

N/A

How was the potential cultural significance of the materials collected in your study to local communities considered in your research design? Were Indigenous peoples and/or local researchers and institutions involved with archaeological excavations / collection of specimens? If so, please provide a description of their involvement.

N/A

If your manuscript includes photographs of human remains please indicate whether authors obtained permission from descendants or affiliated cultural communities to do so.

N/A
