# Supplementary material for: Prevalence of Chlamydia trachomatis and Neisseria gonorrhoeae infections and associated risk factors among pregnant women and key populations in Kenya: A multi-centre cross-sectional study
Source: PLOS Glob Public Health. 2026 Feb 24;6(2):e0005479. doi: 10.1371/journal.pgph.0005479 (PMC12931752; doi:10.1371/journal.pgph.0005479)
Supplement: S1 Table — (DOCX) [file pgph.0005479.s002.docx]

# **S1 Table. Baseline characteristics of the study populations at each study location, February-July 2022.**

|  | **Pregnant Women** | | | **Key Populations** | |
| --- | --- | --- | --- | --- | --- |
| **Characteristic** | **Nairobi ANC (N=301)** | **Mombasa ANC (N=301)** | **Homabay ANC (N=302)** | **Nairobi Dice (N=224)** | **Mombasa Dice (N=224)** |
| **Age (years)** |  |  |  |  |  |
| Mean ±SD | 27.3 ±5.8 | 27.7 ±5.3 | 25.8 ±5.5 | 29.9 ±6.4 | 32.0 ±8.3 |
| Min – Max | 17 – 45 | 18 – 44 | 16 – 47 | 18 – 50 | 17 – 62 |
| **Age groups [n (%)]** |  |  |  |  |  |
| <20 | 16 (5.3) | 7 (2.3) | 30 (9.9) | 4 (1.8) | 8 (3.6) |
| 20-29 | 195 (64.8) | 194 (64.5) | 202 (66.9) | 118 (52.7) | 89 (39.7) |
| 30-39 | 78 (25.9) | 95 (31.6) | 67 (22.2) | 85 (37.9) | 84 (37.5) |
| 40-49 | 12 (4.0) | 5 (1.7) | 3 (1.0) | 16 (7.1) | 36 (16.1) |
| ≥50 | 16 (5.3) | 7 (2.3) | 30 (9.9) | 1 (0.4) | 7 (3.1) |
| **Nationality** **[n (%)]** |  |  |  |  |  |
| Kenyan | 296 (98.3) | 298 (99.0) | 302 (100) | 215 (96.0) | 223 (99.6) |
| Non-Kenyan | 5 (1.7) | 3 (1.0) | 0 (0.0) | 9 (4.0) | 1 (0.4) |
| **Residence [n (%)]** |  |  |  |  |  |
| Urban | 297 (98.7) | 79 (26.2) | 252 (83.4) | 221 (98.7) | 137 (61.2) |
| Rural | 4 (1.3) | 222 (73.8) | 50 (16.6) | 3 (1.3) | 87 (38.8) |
| **Education level [n (%)]** |  |  |  |  |  |
| No education | 0 (0.0) | 3 (1.0) | 0 (0.0) | 1 (0.4) | 1 (0.4) |
| Some primary education | 10 (3.3) | 29 (9.6) | 10 (3.3) | 25 (11.2) | 67 (29.9) |
| Completed primary education | 35 (11.6) | 36 (12.0) | 52 (17.2) | 27 (12.1) | 36 (16.1) |
| Some secondary education | 35 (11.6) | 20 (6.6) | 36 (11.9) | 37 (16.5) | 45 (20.1) |
| Completed secondary education | 108 (35.9) | 99 (32.9) | 101 (33.4) | 55 (24.6) | 52 (23.2) |
| Some tertiary education | 32 (10.6) | 9 (3.0) | 32 (10.6) | 38 (17.0) | 12 (5.4) |
| Completed tertiary education | 81 (26.9) | 105 (34.9) | 71 (23.5) | 41 (18.3) | 11 (4.9) |
| **Occupation [n (%)]** |  |  |  |  |  |
| Salaried | 44 (14.6) | 71 (23.6) | 39 (12.9) | 40 (17.9) | 5 (2.2) |
| Wage-earner | 21 (7.0) | 31 (10.3) | 0 (0.0) | 17 (7.6) | 196 (87.5) |
| Self-employed | 58 (19.3) | 70 (23.3) | 135 (44.7) | 152 (67.9) | 10 (4.5) |
| Unemployed | 145 (48.2) | 121 (40.2) | 84 (27.8) | 2 (0.9) | 8 (3.6) |
| Student | 33 (11.0) | 8 (2.7) | 44 (14.6) | 13 (5.8) | 5 (2.2) |

ANC=antenatal care; Dice=Drop-in-Centre; SD=standard deviation.
