# Supplementary material for: Prevalence of Chlamydia trachomatis and Neisseria gonorrhoeae infections and associated risk factors among pregnant women and key populations in Kenya: A multi-centre cross-sectional study
Source: PLOS Glob Public Health. 2026 Feb 24;6(2):e0005479. doi: 10.1371/journal.pgph.0005479 (PMC12931752; doi:10.1371/journal.pgph.0005479)
Supplement: S2 Table — (DOCX) [file pgph.0005479.s003.docx]

# **S2 Table. Key population-specific baseline characteristics according to the Dice clinic location, February-July 2022.**

| **Characteristic** | **Nairobi (N=224)** | **Mombasa (N=224)** |
| --- | --- | --- |
| **Sex [n (%)]** |  |  |
| Male | 123 (54.9) | 92 (41.1) |
| Female | 101 (45.1) | 127 (56.7) |
| Transgender | 0 (0) | 5 (2.2) |
| **Type of Key population [n (%)]** |  |  |
| FSW | 101 (45.1) | 127 (56.7) |
| MSM | 104 (46.4) | 91 (40.6) |
| MSM-SW | 19 (8.5) | 6 (2.7) |
| **Sexual behaviour in the previous month** |  |  |
| No who reported their sexual behaviour [n (%)] | 127 (56.7) | 205 (91.5) |
| Median (Min – Max) | 1.0 (0 – 60) | 3.0 (1 – 60) |

Dice=Drop-in-Centre; FSW=female sex worker; MSM=men who have sex with men; MSM-SW=men who have sex with men who sell sex; SD=standard deviation.
