# Supplementary material for: Prevalence of Chlamydia trachomatis and Neisseria gonorrhoeae infections and associated risk factors among pregnant women and key populations in Kenya: A multi-centre cross-sectional study
Source: PLOS Glob Public Health. 2026 Feb 24;6(2):e0005479. doi: 10.1371/journal.pgph.0005479 (PMC12931752; doi:10.1371/journal.pgph.0005479)
Supplement: S3 Table — (DOCX) [file pgph.0005479.s004.docx]

# **S3 Table. Obstetrical history of pregnant women according to the ANC clinic location, February-July 2022.**

| **Characteristic** | **Nairobi (N=301)** | **Mombasa (N=301)** | **Homabay (N=302)** |
| --- | --- | --- | --- |
| **Gestational age (weeks)** |  |  |  |
| Mean ±SD | 28.4 ±7.6 | 26.6 ±8.6 | 25.1 ±8.5 |
| Min – Max | 5 – 42 | 4 – 40 | 5 – 42 |
| 1st trimester [n (%)] | 17 (5.6) | 29 (9.6) | 38 (12.6) |
| 2nd trimester [n (%)] | 122 (40.5) | 125 (41.5) | 154 (51.0) |
| 3rd trimester [n (%)] | 162 (53.8) | 147 (48.8) | 110 (36.4) |
| **Pregnancies characteristics** |  |  |  |
| No of pregnancies, including the current one (mean ±SD) | 2.2 ±1.3 | 2.2 ±1.3 | 2.5 ±1.6 |
| Number of women with a previous live birth [n (%)] | 174 (57.8) | 157 (52.2) | 195 (64.6) |
| Number of live births (mean ±SD) | 1.0 ±1.1 | 0.9 ±1.1 | 1.3 ±1.4 |
| Number of women with pregnancy loss [n (%)] | 46 (15.3) | 66 (21.9) | 34 (11.3) |
| Number of loss (mean ±SD) | 0.2 ±0.5 | 0.3 ±0.6 | 0.2 ±0.5 |

ANC=antenatal care; SD=standard deviation.
