# Supplementary material for: Prevalence of Chlamydia trachomatis and Neisseria gonorrhoeae infections and associated risk factors among pregnant women and key populations in Kenya: A multi-centre cross-sectional study
Source: PLOS Glob Public Health. 2026 Feb 24;6(2):e0005479. doi: 10.1371/journal.pgph.0005479 (PMC12931752; doi:10.1371/journal.pgph.0005479)
Supplement: S4 Table — (DOCX) [file pgph.0005479.s005.docx]

# **S4 Table. History of STIs in the study populations at each study location, February- July 2022.**

|  | **Pregnant Women** | | | **Key Populations** | |
| --- | --- | --- | --- | --- | --- |
| **Characteristic** | **Nairobi ANC (N=301)** | **Mombasa ANC (N=301)** | **Homabay ANC (N=302)** | **Nairobi Dice (N=224)** | **Mombasa Dice (N=224)** |
| **n (%) of participants reporting a history of STI** | 14 (4.7) | 8 (2.7) | 13 (4.3) | 68 (30.4) | 21 (9.4) |
| **Previous STI [n (%)]** |  |  |  |  |  |
| HIV infection/ AIDS | 13 (4.3) | 0 | 13 (4.3) | 59 (26.3) | 0 |
| Syphilis | 1 (0.3) | 1 (0.3) | 0 | 2 (0.9) | 0 |
| Gonorrhoea | 0 | 0 | 0 | 6 (2.7) | 3 (1.3) |
| Genital Herpes | 0 | 0 | 0 | 0 | 1 (0.4) |
| Sexually transmitted disease (not specified) | 0 | 7 (2.3) | 0 | 2 (0.9) | 17 (7.6) |

AIDS= acquired immunodeficiency syndrome; HIV= human immunodeficiency virus; STI= sexually transmitted infection.
