# Supplementary material for: Prevalence of Chlamydia trachomatis and Neisseria gonorrhoeae infections and associated risk factors among pregnant women and key populations in Kenya: A multi-centre cross-sectional study
Source: PLOS Glob Public Health. 2026 Feb 24;6(2):e0005479. doi: 10.1371/journal.pgph.0005479 (PMC12931752; doi:10.1371/journal.pgph.0005479)
Supplement: S5 Table — (DOCX) [file pgph.0005479.s006.docx]

# **S5 Table. Treatments given to pregnant women with positive NG and/or CT diagnostic tests at each ANC location, February-July 2022.**

| **Treatment received** | **Nairobi (N=301)** | **Mombasa (N=301)** | **Homabay (N=302)** | **Overall (N=904)** |
| --- | --- | --- | --- | --- |
| **For NG only positive pregnant women [n (%)]** |  |  |  |  |
| Ceftriaxone | 1 (50.0) | 0 (0) | 0 (0) | 1 (16.7) |
| Azithromycin + cefixime^1^ | 0 (0) | 1 (100) | 2 (66.7) | 3 (50.0) |
| Not treated (patient lost to follow-up) | 1 (50.0) | 0 (0) | 1 (33.3) | 2 (33.3) |
| **For CT only positive pregnant women [n (%)]** |  |  |  |  |
| Azithromycin^1^ | 29 (87.9) | 18 (94.7) | 20 (62.5) | 67 (79.8) |
| Erythromycin^1^ | 3 (9.1) | 0 (0) | 1 (3.1) | 4 (4.8) |
| Doxycycline | 0 (0) | 0 (0) | 1 (3.1) | 1 (1.2) |
| Azithromycin1 + other^2^ | 0 (0) | 0 (0) | 9 (28.1) | 9 (10.7) |
| Not treated (patient lost to follow-up) | 1 (3.0) | 1 (5.3) | 1 (3.1) | 3 (3.6) |
| **For CT and NG positive pregnant women [n (%)]** |  |  |  |  |
| Azithromycin | 1 (100) | 0 (0) | 0 (0) | 1 (33.3) |
| Azithromycin + cefixime^1^ | 0 (0) | 0 (0) | 2 (100) | 2 (66.7) |

ANC=antenatal care; CT=*Chlamydia trachomatis*; NG=*Neisseria gonorrhoeae*.

^1^ Standard of care treatment for pregnant women in Kenya.

^2^ Eight participants at Homabay received metronidazole for treatment of CT, according to their previous standard of care and 1 received cefixime orally with clindamycin phosphate +clotrimazole vaginal suppository.
