# Supplementary material for: Prevalence of Chlamydia trachomatis and Neisseria gonorrhoeae infections and associated risk factors among pregnant women and key populations in Kenya: A multi-centre cross-sectional study
Source: PLOS Glob Public Health. 2026 Feb 24;6(2):e0005479. doi: 10.1371/journal.pgph.0005479 (PMC12931752; doi:10.1371/journal.pgph.0005479)
Supplement: S6 Table — (DOCX) [file pgph.0005479.s007.docx]

# **S6 Table. Treatments given to key populations with positive NG and/or CT diagnostic tests at each Dice location, February-July 2022.**

| **Treatment received** | **Nairobi (N=224)** | **Mombasa (N=224)** | **Overall (N=448)** |
| --- | --- | --- | --- |
| **For NG only positive key population [n (%)]** |  |  |  |
| Azithromycin + ceftriaxone^1^ | 17 (85.0) | 13 (100) | 30 (90.9) |
| Not treated (patient lost to follow-up) | 3 (15.0) | 0 (0) | 3 (9.1) |
| **For CT only positive key population [n (%)]** |  |  |  |
| Doxycycline^1^ | 14 (77.8) | 23 (100) | 37 (90.2) |
| Not treated (patient lost to follow-up) | 4 (22.2) | 0 (0) | 4 (9.8) |
| **For CT and NG positive key population [n (%)]** |  |  |  |
| Azithromycin + ceftriaxone^1^ | 4 (66.7) | 1 (33.3) | 5 (55.6) |
| Ceftriaxone + doxycycline^1^ | 0 (0) | 2 (66.7) | 2 (22.2) |
| Not treated (patient lost to follow-up) | 3 (33.3) | 0 (0) | 2 (22.2) |

CT=*Chlamydia trachomatis*; Dice=Drop-in-centre; NG=*Neisseria gonorrhoeae*.

^1^ Standard of care treatment for key population in Kenya. Azithromycin is recommended for the treatment of CT when a patient has concomitant gonorrhoea infection and has been treated with ceftriaxone.
