# Supplementary material for: Prevalence of Chlamydia trachomatis and Neisseria gonorrhoeae infections and associated risk factors among pregnant women and key populations in Kenya: A multi-centre cross-sectional study
Source: PLOS Glob Public Health. 2026 Feb 24;6(2):e0005479. doi: 10.1371/journal.pgph.0005479 (PMC12931752; doi:10.1371/journal.pgph.0005479)
Supplement: S7 Table — (DOCX) [file pgph.0005479.s008.docx]

# **S7 Table. Prevalence and prevalence ratios for NG and/or CT according to demographics in pregnant women** **in Kenya, February-July 2022.**

| **Variable** | **Unadjusted prevalence (95%CI)** | **Unadjusted PR (95%CI)** | **Adjusted prevalence (95%CI)** | **Adjusted PR (95%CI)** |
| --- | --- | --- | --- | --- |
| **Residence** |  |  |  |  |
| Rural | 7.2 (4.5-11.0) | 0.62 (0.39-1.00) | 8.3 (5.0-13.5) | 0.86 (0.46-1.61) |
| Urban | 11.6 (9.2-14.4) | Ref | 9.6 (7.2-12.7) | Ref |
| **Region** |  |  |  |  |
| Homabay | 12.3 (8.8-16.5) | Ref | 10.4 (7.3-14.6) | Ref |
| Mombasa | 6.6 (4.1-10.1) | 0.54 (0.32-0.91) | 6.9 (4.1-11.3) | 0.66 (0.35-1.27) |
| Nairobi | 12.0 (8.5-16.2) | 0.98 (0.64-1.51) | 10.7 (7.3-15.5) | 1.03 (0.65-1.62) |
| **Age** |  |  |  |  |
| >20 | 18.9 (9.4-32.0) | 1.57 (0.86-2.86) | 17.1 (9.2-29.4) | 1.45 (0.77-2.71) |
| 20-29 | 12.0 (9.5-14.9) | Ref | 11.7 (9.3-14.6) | Ref |
| 30-39 | 4.6 (2.3-8.1) | 0.38 (0.21-0.71) | 4.6 (2.5-8.1) | 0.39 (0.21-0.73) |
| 40-49 | 5.0 (0.1-24.9) | 0.42 (0.06-2.84) | 4.5 (0.6-26.1) | 0.39 (0.06-2.60) |
| **Trimester**1 |  |  |  |  |
| 1 | 8.3 (3.4-16.4) | 0.85 (0.39-1.83) | 7.2 (3.4-14.7) | 0.81 (0.38-1.73) |
| 2 | 11.2 (8.3-14.7) | 1.14 (0.77-1.71) | 9.9 (7.3-13.3) | 1.09 (0.72-1.65) |
| 3 | 9.8 (7.1-13.1) | Ref | 9.0 (6.6-12.2) | Ref |

Adjusted PR from logistic regression. Adjusted PR was only computed for participants with NG or CT where the number of cases was sufficient for model convergence. CI= confidence interval; CT= *Chlamydia trachomatis*; NG= *Neisseria gonorrhoeae*; PR= prevalence ratio; Ref= reference level.
