# Supplementary material for: Prevalence of Chlamydia trachomatis and Neisseria gonorrhoeae infections and associated risk factors among pregnant women and key populations in Kenya: A multi-centre cross-sectional study
Source: PLOS Glob Public Health. 2026 Feb 24;6(2):e0005479. doi: 10.1371/journal.pgph.0005479 (PMC12931752; doi:10.1371/journal.pgph.0005479)
Supplement: S8 Table — (DOCX) [file pgph.0005479.s009.docx]

# **S8 Table. Prevalence and prevalence ratios for NG and/or CT according to demographics in the key populations in Kenya, February-July 2022.**

| **Variable** | **Unadjusted prevalence (95%CI)** | **Unadjusted PR (95%CI)** | **Adjusted prevalence (95%CI)** | **Adjusted PR (95%CI)** |
| --- | --- | --- | --- | --- |
| **Residence** |  |  |  |  |
| Rural | 13.3 (7.1-22.1) | 0.67 (0.38-1.18) | 12.3 (6.6-21.8) | 0.67 (0.34-1.33) |
| Urban | 19.9 (15.9-24.4) | Ref | 18.8 (14.8-23.5) | Ref |
| **Region** |  |  |  |  |
| Mombasa | 17.5 (12.7-23.1) | 0.89 (0.60-1.31) | 17.8 (13.0-24.0) | 1.05 (0.65-1.71) |
| Nairobi | 19.6 (14.7-25.5) | Ref | 16.8 (12.1-22.8) | Ref |
| **Age** |  |  |  |  |
| <20 | 66.7 (34.9-90.1) | 3.05 (1.90-4.91) | 66.3 (37.0-86.8) | 3.00 (1.39-6.44) |
| 20-29 | 21.8 (16.4-28.1) | Ref | 21.8 (16.6-28.1) | Ref |
| 30-39 | 14.8 (9.8-21.1) | 0.68 (0.43-1.06) | 14.5 (9.9-20.7) | 0.67 (0.41-1.10) |
| 40-49 | 9.6 (3.2-21.0) | 0.44 (0.18-1.05) | 8.3 (3.5-18.5) | 0.38 (0.15-0.97) |
| ≥50 | 0 |  |  |  |
| **Type of key population** |  |  |  |  |
| FSW | 18.1 (13.3-23.7) | Ref | 18.0 (13.3-23.8) | Ref |
| MSM | 19.0 (13.7-25.2) | 1.05 (0.70-1.57) | 16.4 (11.7-22.5) | 0.92 (0.58-1.45) |
| MSM-SW | 20.0 (6.8-40.7) | 1.11 (0.48-2.54) | 18.5 (7.7-38.4) | 1.03 (0.40-2.66) |

Adjusted PR from logistic regression. Adjusted PR was only computed for participants with NG or CT where the number of cases was sufficient for model convergence. CI= Confidence Interval; CT= *Chlamydia trachomatis*; FSW= female sex worker; MSM= men who have sex with men; MSM-SW= men who have sex with men who sell sex; NG= *Neisseria gonorrhoeae*; PR= prevalence ratio; Ref= reference level.
